# Strategies for enhancing participation of underrepresented patient populations in cancer clinical trials: learning from “bright spots”

**DOI:** 10.1186/s12885-026-16181-1

**Published:** 2026-06-13

**Authors:** Jennifer E. Miller, Jason Schwartz, Reshma Ramachandran, Joseph Ross, Sakinah C. Suttiratana

**Affiliations:** Yale School of Medicine, Yale University New Haven, New Heaven, CT USA

## Abstract

**Background:**

Adequate participation of women, older adults, racial and ethnic subgroups, and other groups, in pivotal trials for cancer therapies is critical for understanding and trusting the safety and efficacy of new medical products across populations. Despite consensus on the importance of representative research and extensive documentation of trial participation barriers, progress has been minimal for most groups, raising the question: What more can be done to enhance participation of underrepresented patient populations in cancer clinical trials, and in turn, improve medical evidence?

**Methods:**

We used a “bright spot” approach to understand what drives successful representation of Black and LatinX patients in cancer clinical trials. We conducted 1-h interviews with “bright spot trial” teams, teams who conducted trials that adequately enrolled Black or LatinX patients relative to disease burden among pivotal trials supporting FDA approval of novel cancer therapeutics between 2012 and 2021, to identify organizational contexts and facilitators associated with success across multiple action levels. Transcripts were analyzed using constant comparative methods.

**Results:**

Thirteen participants from 8 bright spot trial teams described a coherent set of strategies associated with representative enrollment, clustering across five levels: patient and community, clinician, trial and site, sponsor organization, and policy. Key practices included prospective budgeting for mitigating participant expenses; patient- and community co-developed educational programs; navigator use; early clinician engagement and referral training; protocol simplification, decentralization, and broadening of eligibility criteria; expansion into community and satellite hospitals affiliated with NCI Designated Cancer Center trial sites; and sustained, non-transactional community partnerships. At the organizational level, “bright spot trial” teams emphasized the importance of executive leadership commitment, real-time dashboarding on enrollment goals, performance-based incentives, employee training, and minimizing reliance on generic vendor solutions.

**Conclusions:**

Sponsors that achieved representative enrollment in oncology trials shared a common operational playbook: reduce patient and site burden, expand access to where care is delivered, widen trial eligibility, invest in trusted relationships, and embed accountability through leadership, data and incentives. Translating these strategies from bright spots into standard practice, supported by clearer regulatory guidance and evaluation, offers a concrete path toward more accessible and generalizable cancer clinical research.

**Supplementary Information:**

The online version contains supplementary material available at 10.1186/s12885-026-16181-1.

## Introduction

Ensuring adequate representation of women, older adults, racial and ethnic minorities, and other groups, among patients enrolled in pivotal trials of cancer therapies is critical for understanding and having confidence in the safety and efficacy of new medical products across distinct patient populations [1–6]. Despite broad consensus on its importance and sustained investments in improving practices, measurable progress has been limited for many groups [7]. For example, we previously analyzed 121 trials supporting FDA approval of 111 cancer therapeutics from 2012 through 2021, finding zero trials that adequately represented the sex, age, race, and ethnicity of patients in comparison to either the US patient population with each approved indication or US census data. Further, we found no improvements in representation by sex, age, race or ethnicity, over the 10-year period (*P* values > 0.05) [8]. Trial participation rates, in general, remain low for adults with cancer, estimated at about 7% [9].

The intractability of this problem raises the question: What more can be done to address participation disparities and, in turn, improve medical evidence [10, 11]? While barriers to trial participation are well documented, evidence-based strategies to overcome them are understudied. [12] Reviewing 65 articles published between 1966 and 2005, Ford and colleagues, for instance, identified 150 distinct barriers to accrual in cancer trials, with mistrust of research and the medical system most frequently cited [13]. In contrast, when we reviewed leading guidance published by the FDA, European Medicines Agency, World Health Organization, and other key groups, we found 14 key strategies to enhance representation. These included broadening eligibility criteria, using enrollment targets that reflect disease prevalence, and engaging patients in trial design [14]. Though reasonable, recommendations were often vague and lacked supportive evidence [15].

To bridge these knowledge gaps and generate evidence on what works, we applied a “bright spot” or “positive deviance” approach, to identify and study sponsors who have succeeded, despite facing similar constraints as others, in enrolling representative patient groups in cancer-related pivotal trials. The bright spot approach assumes that at least one person or organization in a community has determined how to solve a problem confounding others and that sharing those successful strategies with the wider community can produce similar results [16]. The bright spot approach has improved outcomes in diverse areas of public health and healthcare delivery [17], from reducing MRSA infection rates in Acute Care Medical Centers and Nursing Homes [18] to enhancing HIV care retention [19]. In all cases, top performers are identified and interviewed to characterize facilitators for success [20–25].

In our prior work described above, analyzing pivotal trials supporting FDA approval of novel cancer therapeutics between 2012 and 2021, we identified 33 “bright spot trials,” trials that achieved adequate enrollment of Black or LatinX-identifying patients relative to disease burden [26]. Here, we report findings from qualitative interviews with the organizations sponsoring those bright spot trials. We describe the organizational contexts, specific practices, and shared strategies of high-performing sponsors to inform actionable evidence-based efforts that can strengthen representation in cancer clinical research and move beyond reiterating barriers.

## Methods

### Eligibility and recruitment

Our previously published study examined 121 pivotal trials supporting FDA approval of 111 novel cancer therapeutics between 2012 and 2021 to find “bright spot” trials, defined as trials that adequately represented Black or LatinX identifying patients in proportions ≥ 80% of their representation among the US patient populations with each targeted indication, using 2016 US Cancer Statistics (e.g., if 20% of the US patient population with the targeted indication identifies as LatinX, the trial benchmark would be ≥ 16%) [26]. This study extends our prior study and completes the first two steps characterized by the Bradley et al. bright spot methodology: 1) identify “bright spot” organizations that consistently demonstrate exceptionally high performance in the area of interest and 2) study the organizations using qualitative methods to generate hypotheses about enabling top performance [17].

We sent personalized, professional electronic correspondence to key staff and administrators involved in each identified bright spot trial sponsored by a large company (50 largest by 2016 market capitalizations) to schedule individual or group interviews. Individuals were identified from each trial’s registration on clinicaltrials.gov and with help from each sponsor’s director of diversity, population health, health equity, medical affairs, or bioethics. Potential participants who did not respond to the first invitation received two reminders by email encouraging participation. All participants were adults over the age of 21.

This collection of information was approved by the Office of Management and Budget (OMB) under the Paperwork Reduction Act. The OMB control number is 0910–0847 and the expiration date is 02/28/2026.

### Human ethics and consent to participate declaration

This study was reviewed by and received an exemption from the Institutional Review Board (IRB) at the Yale School of Medicine as participants responded to questions about their professional workplaces. Every human participant was emailed a copy of our “Oral Consent Email Script,” which was IRB reviewed, explaining the rationale, that there were no known risks, critical information about study participation, and intent to publish findings. Participants were informed again before the start of interviews that, “all information obtained about you during the conversation will be held in confidence and accessible only to the research team of this project…Data will be anonymized and aggregated for reporting purposes.” Participants were also informed that findings from this study would be characterized and disseminated through peer reviewed journal articles. The consent script was reviewed again before beginning any interviews and or surveys. Each participant provided oral informed consent and was given an opportunity to ask any questions about the study, before completing an electronic survey about their organization and practices and 60-min virtual interviews, grouped by sponsor, on efforts to field their representative clinical trials. A copy of the consent script and interview guide are available in the Appendix. This study adheres to the Declaration of Helsinki.

### Data collection and analysis

Qualitative methods were selected as appropriate to identify factors that explain how sponsor and trial environments influence clinical trial operations and facilitate representative trials. Positive deviance or bright spot approaches assert that lessons may be learned from those excelling under unfavorable or current state conditions. Descriptors of individuals and bright spot trial sponsors have been excluded to minimize unwanted disclosure and to balance transparency and a range of confidentiality preferences.

Descriptive characteristics about interviewed participants and their organizations were captured using a standardized data collection form in Qualtrics. Data about the trial context and conditions were collected during semi-structured, qualitative interviews covering clinical trial practices, strategies for patient recruitment and representativeness, and organizational characteristics. All interviews were conducted by the first author of this paper (JM) with experience in industry-sponsored clinical trials and conducting qualitative interviews. The interview guide, which was developed for this study, began with a “grand tour” question that all respondents should have been able to answer with ease. The opening questions set the stage for the general areas of inquiry during the interview before getting into more detailed descriptions of activities within clinical trial sponsor organizations. Example questions from the interview guide are provided in Table 1 and the full interview guide is available in the Supplemental Appendix. Interviews were audio-recorded and transcribed by Zoom. Zoom transcripts were reviewed and verified by the interviewer and a member of the research team. After transcriptions were verified, audio recordings were deleted.

**Table 1:** Example questions from the semi-structured interview guide

• In the last 3–5 years, what are the major initiatives your group has undertaken to enable representation of women, older adults, and or Asian, Black and LatinX identifying patients in trials?
• Can you describe instances of difficulty and success with these initiatives? Why did initiatives work or not work?
• Can you describe how these initiatives got started (i.e., origin of idea, funding, partners)?
• More generally, can you describe instances of success in recruiting and retaining women, older adults, and racial and ethnic minoritized patients in trials?
• Can you describe instances of difficulty in recruiting and retaining women, older adults, and racial and ethnic minoritized patients in trials and how these difficulties were overcome?
• What systemic barriers does your company or the research ecosystem encounter in increasing demographic participation (e.g., incentives, culture)? Does your company work to overcome these barriers, if so how or if no, why not?
• What type of support do you think patients (women, older adults and or racial and ethnic minoritized patients) need to increase their trial participation? Does your company work to provide this support, if so how or if no, why not?

The research team used a pragmatic, team-based approach to qualitative analysis such that a blend of inductive and deductive analysis was combined with broader systems-level conceptual framing in order to deliver practicable study insights. Adapting techniques from the framework analysis, the interviewer (first author of this paper) and the last author of this paper, both experienced in qualitative research, became familiar with the data and analyzed transcripts using a grounded theory approach, constant comparative analysis methods, and considering relevant literature. [27, 28] A coding scheme was developed after the first two interviews and iterated for subsequent interviews. Two researchers independently analyzed and coded all transcripts and met to discuss categorization challenges and achieve consensus. Analysis discussions were sometimes also attended by other members of the research team who posed questions about the data based on their multidisciplinary expertise and the focal research questions. Representative quotes were selected for each code and any analytic discrepancies reconciled in group meetings. Codes related to facilitators were organized by levels of action to situate respondent ideas within a broader arena of clinical trials research. Analyzing across data, coding syntheses were refined by four members of the research team who used initial coding, level of actions classifications, and group consensus discussions to refine analytic interpretations of the data. Despite not interviewing members from all large bright spot companies, key concept saturation was achieved in the sample such that no new categories of ideas were noted by the eighth interview. Conceptual saturation means that the research team believes it has captured the key concepts related to achieving representative clinical trials among large companies who have sponsored bright spot trials.

## Results

### Sample characteristics

Our previous study identified 31 unique bright spot trials (sponsored by 23 organizations), 19 of which were sponsored by a large pharmaceutical company (*n* = 13 companies). Eight (62%) of the 13 large companies participated in this study, with a mean of 1.6 individuals per company and a total of 13 participants. Interviews lasted a mean of 55 min (range, 30–81).

### Strategies and enablers of representative oncology clinical trials

**Fig. 1:**
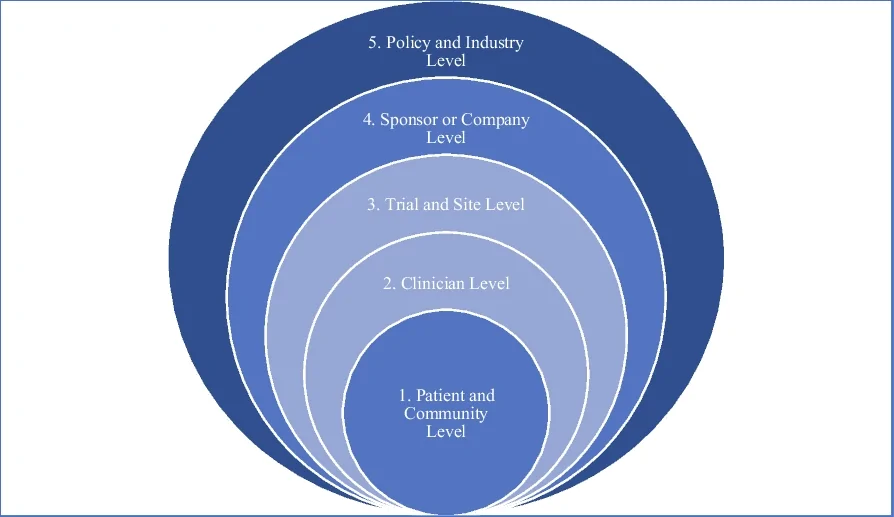
Levels of action to advance representation in clinical research: A Multilevel Framework

**Fig. 2:**
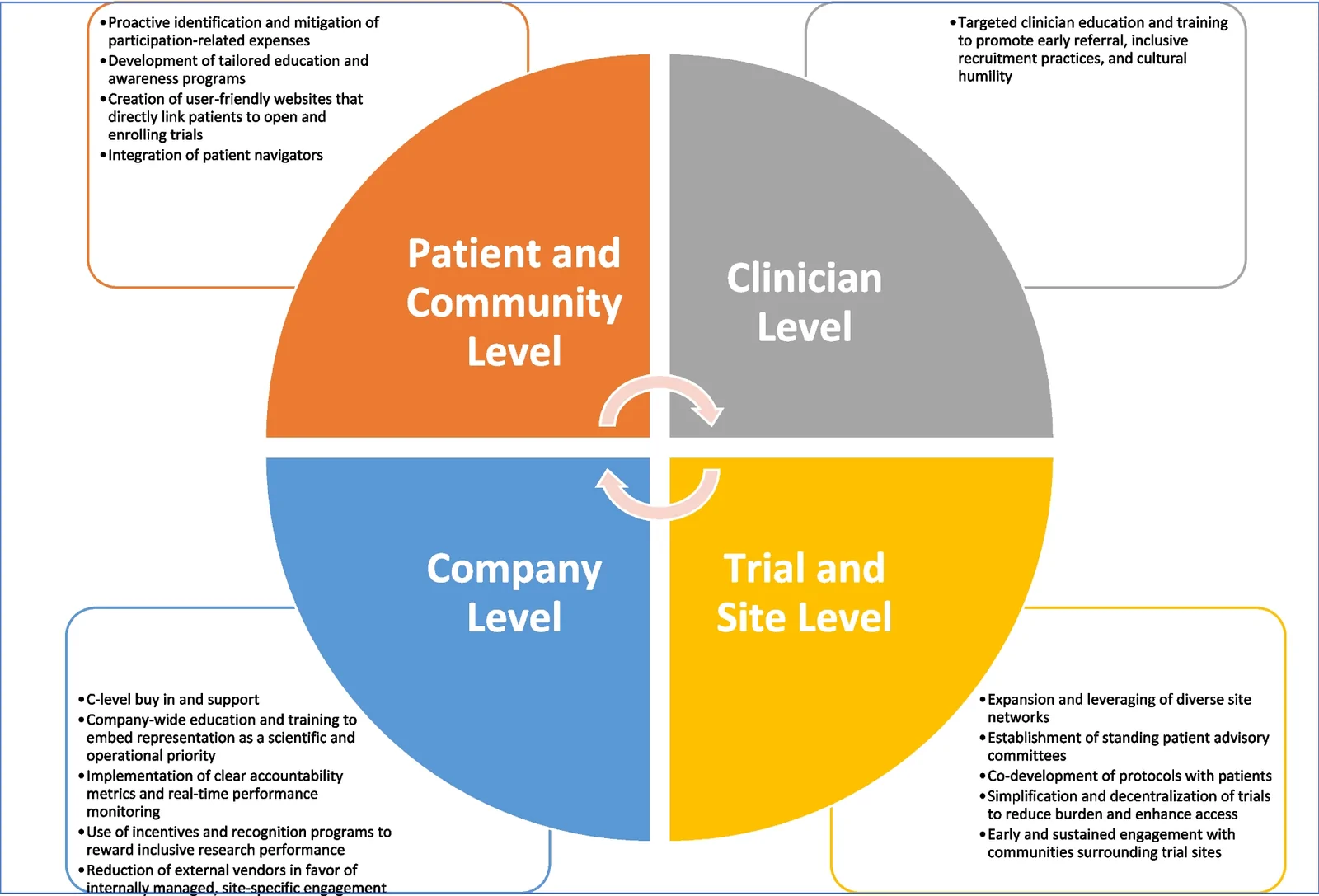
Multi-level Framework of Facilitators for Representative Research

Bright spot trial teams described 14 successful strategies for enhancing participation of patient groups in cancer-related pivotal trials, which correspond to actions on the (1) patient/community, (2) clinician, (3) trial/trial site, (4) sponsor/company, and (5) policy levels. (Figs. 1 and 2).

### Patient and community level facilitators

On the patient and community levels, sponsors shared 4 methods for advancing representative clinical research: identifying and remedying financial barriers to trial participation, developing trusted patient and community-driven educational programs, redesigning their websites to directly connect qualifying patients with enrolling trial sites, and working with health navigators to support patients through diagnosis and enrollment. (Fig. 2, Table 2).

**Table 2:** Patient and Community Level Strategies and Enablers for Representative Research Used by Bright Spots

Facilitators	Study-Derived Interpretative Conceptualization	Exemplar Quotes
Expense mitigation	Bright spots have processes to identify and remedy participant expenses. Trial teams build identified expenses into study budgets as line items of support	• “We have a…study budget line item that supports patients who need extra support… to participate…. We're reviewing study budgets with an eye toward providing more out of pocket expense support for patients.” • “We give people a debit card. We cover their costs. Sometimes we pay for a family member to travel too if needed.” • “We use Greenphire ClinCard for reimbursements… because you don’t have to put money up front.”
Education	Bright spots invest in multipronged educational programs for patients and the public Programs provide primers on, explain the value of, and address patient fears about research participation Programs are codeveloped or developed with insights from patients. Sometimes materials are also developed by cancer centers with support from bright spots Programs and materials are often distributed by referring clinicians, sites, community organizations, and navigators	• “I think the 1 st one is they need to know about it (research) right? So they need to know that there's an option… need the ability to have someone have a conversation with them. And wouldn't it be lovely if they already stepped into that doctor's office and already had some foundational awareness of what a clinical trial was. Right. It wasn't like the 1 st time they're hearing about this scary clinical trial. So I think doing our part to raise awareness what we call above study, just like clinical trials, 101 like what is a clinical trial.” • “So I think access and awareness. But the education piece and I think (our company) is doing a really nice job with a lot of our field colleagues and our commercial colleagues at raising, helping, raise the awareness and the story and the messaging around clinical trials as an option.” • “There's a lot of work that needs to be done before you lift off the protocol … which is creating those educational materials to reduce fear around clinical trials. Fear of being treated like a guinea pig, fear the placebo and working with referral physicians on why, inclusion's important and referral earlier is important.” • “We've had community advisory boards help tailor our educational materials to be culturally relevant. This ensures that patients see themselves in the materials and can relate to the stories.” • “We have these regional health equity symposia; XX Cancer center is one of our alliance sites. They submit proposals for these webinars which are very intentionally focused on Latino or Latina education, on the need to go in and get diagnosed.” • “So, a lot of that is through the sites. I think what we are trying to do is partner with sites to distribute that, so that we are not necessarily reaching out purely as a sponsor, but actually based on health, education sites. Or you know what we have also discussed is community engagement through navigation, through nurse navigation…. we're kind of trying to share with partners rather than directly from (our company).”
Websites appropriately connect patients to open trials	Bright spots re-design their websites to directly connect patients with enrolling clinical trial sites for which they will likely qualify for enrollment	• “We actually had to hire a company to do an overlay to make it (the website) easier to use… at that point, the website wasn't actually connecting people to sites, because there had been some input from sites that they were getting inundated by with calls from people who really, would not qualify • So, we put a little pre-screener (on the website), and then you get through the prescreen, and then you actually get the site info. We had to go back to the sites and say, hey, I know you originally said this, but it's really not that helpful for somebody just to get a name. They don't even have the contact. It's it just adds to the energy and the stress of the whole situation. All of the sites ended up agreeing, yes, that's fine and it became much more user friendly.”
Navigators and community health workers	Bright spots work with community health workers and navigators, perceiving them as trusted messengers for patients considering trial participation The role of navigators are multipronged and included, helping patients navigate the health system and become aware of, access and afford medical tests, diagnoses, trials, treatments, and care Bright spots invest in strengthening the infrastructure for navigators and community health workers, recognizing gaps in their abilities to support clinical trials	• “Essentially, they’re community, they’re patient navigators but in the community. So, they’re lay navigators, but they're trained. They usually have some kind of public health background…. They tend to be a public health educator in the Latino community. … they might be working with churches… have Spanish native speakers. We have a retinal specialist who identifies as Black in San Antonio, Texas, but speaks Spanish, he has very good Latina representation.” • “Our healthcare system's a little broken, right? You have limited time with your patient, and you don't always have an advocate in your institution to help people. So, people get a little lost in the system.” • “I have stories from lay navigators in Alabama who are like, you know, it takes someone a year to get talked into going to get cancer screening because they're scared of what they're going to find, and I have to go with them. Hold their hand.” • “A lot of folks just don't know how …(and) where to go, that's why x patient group and x patient navigator (in) communities are so important because they're not just helping people understand that you should get screened, they have social and financial networks to help refer patients. But folks don't know where to go, or they have 2 jobs, and they can't take time off work.” • “There was a great study published at ASCO… where they had real ties between timeliness to help a patient… from symptom onset to diagnosis, from diagnosis to first treatment, from first treatment to…navigation touch points and outcomes were improved every time you could decrease the time to the change.” • “So… talking to various groups of racial and ethnic minorities, the common feedback is not that they didn't want to participate, or were afraid to participate, or didn't understand the study, but mostly that it was not offered to them. That's the part that we're really trying to focus on making it accessible. You still need that component of extra support, you know. At a site and at a patient level, especially like nurse navigators, we're learning, they play a very important role….” • “So one of the things we've done is we've created materials for patients that are culturally tailored and that are developed in partnership with trusted organizations like BlackDoctor.org, Black Health Matters, and Hispanic Communications Network. And that's to be able to provide education that resonates with the intended audience. And we work with them to decide what's the best format. Should it be social media? Should it be a flyer? Should it be a video?” • “So, most of the time when we've, you know, done the direct assessment… nurse navigators… play a very important role, and we've supported a third-party grant program through the American Cancer Society, which seems to have been very, very successful…. We support, I think, 14 of the grants and basically provides not only for the head count for the nurse navigator…but also the sort of community of support and education, and ensuring that those individuals that are placed in those roles have the right resources and tools to support patients and provide that extra level of support throughout this study course. Not just, you know, once they're enrolled, but also for adherence purposes… requirements of the protocol and nurse navigator role is a very, very important role. Which is becoming clearer to us and our desire to try to provide that resource across oncology research centers.” • “We've developed a navigation program to support decentralized trials and patient support… we hired a full-time bilingual patient navigator who can support patients in navigating the logistics, transportation, visit reminders.” • “We funded the hiring of community health workers in partnership with federally qualified health centers and trial sites… who are trained to provide education about trials and refer patients who are potentially eligible,”

### Identification and line-item budgeting for participant-related expenses

There was unanimous use among bright spots of formal processes to identify and remedy trial participation barriers, particularly financial barriers. Common trial participant barriers, described by bright spots, included hotel, transportation, dependent care, and meal related costs along with multiple required site visits correlating with lost wages from participation. Trial teams described building such costs directly into their study budgets, at the outset of each trial, as specific line items of support. Some bright spots also allocated funding for the costs of family members to support a patient’s trial participation. Bright spots also provided prepaid debit cards to patients for certain expenses to prevent patients from having to front money or submit burdensome reimbursement documentation.

### Co-developed educational and awareness programs

Bright spots develop and deliver patient and public facing educational programs about clinical research to increase awareness and participation opportunities, led by dedicated internal teams and informed by or co-developed with patient advisors. Bright spots see these programs as part of their responsibility to provide patients and the public with clinical trial primers:

*“they need to know…that there's a (research) option…. wouldn't it be lovely if they already stepped into that doctor's office and… had some foundational awareness of what a clinical trial was…. It wasn't like the 1 st time they're hearing about this scary clinical trial. So I think doing our part to raise awareness…clinical trials 101…*

Some educational programs also describe the value of research for society. One company described an initiative to fund The Center for Information and Study on Clinical Research Participation (CISCRP) to develop a video depicting what could happen in the absence of clinical trial support and participation. The video showed empty pharmacy shelves and aimed to increase access to trials through enhanced public awareness of trials as an option:

*“I love the video, the empty pharmacy. I think* CISCRP *and another sponsor did together. It's such a powerful message that if clinical trials didn't exist... This pharmacy would be empty, like the things that you take over the counter all had to go through some form of clinical research…I think (our company) is doing a really nice job… at raising… awareness and…messaging around clinical trials as an option.”*

Educational programs also aim to address patients’ fears about research participation and build trust in the research process: *“There's a lot of work that needs to be done before you lift off the protocol … which is creating those educational materials to reduce fear around clinical trials. Fear of being treated like a guinea pig, fear the placebo….”*

Programs are co-developed with, or with insights from, patients and community partners and tailored to specific communities. Patient input was described as particularly important for matching patient literacy and language needs along with delivery mechanisms. Educational programs are generally audience-specific, employ culturally competent strategies, and are available in multiple languages and literacy-appropriate levels:* “We've had community advisory boards help tailor our educational materials to be culturally relevant. This ensures that patients see themselves in the materials and can relate to the stories.”*

Sometimes educational materials are developed by cancer centers with support from bright spots. Cancer centers or sites were described as submitting funding requests from bright spots to develop their own patient facing webinars to meet their patient population needs. These webinars tend to educate on the importance of getting screened for certain conditions and how and where to do these screenings. A bright spot trial team, for example, shared that: *“We have these regional health equity symposia; XX Cancer center is one of our alliance sites. They submit proposals for these webinars which are very intentionally focused on Latino or Latina education, on the need to go in and get diagnosed.”*

Educational programs and materials are largely distributed by referring clinicians, trial sites, and trusted community organizations, not the bright spot itself. Patient/lay and nurse navigators are also instrumental in distributing programs:

*“So, a lot of that is through the sites. I think what we are trying to do is partner with sites to distribute that, so that we are not necessarily reaching out purely as a sponsor, but…based on health education… we're kind of trying to share with partners rather than directly from (our company).”*

### Website directly connects patients with applicable enrolling sites

Some bright spots invested in re-designing their website to directly connect patients to open and enrolling trial sites and offer prescreening for eligibility. Previously websites provided site names but did not facilitate a direct connection. Such investments were made at the suggestion of patient groups.

*“We…had to hire a company to…make it (the website) easier to use… at that point, the website wasn't actually connecting people to sites, because there had been some input from sites that they were getting inundated by with calls from people who really, would not qualify…*.*So, we put a little pre-screener (on the website), and then you get through the prescreen, and then you actually get the site info. We had to go back to the sites and say, hey, I know you originally said this, but it's really not that helpful for somebody just to get a name. They don't even have the contact. It's it just adds to the energy and the stress of the whole situation. All of the sites ended up agreeing, yes, that's fine and it became much more user friendly.”*

### Navigators and community health workers

Bright spots work closely with patient, nurse, and lay navigators and community health workers. Navigators were described as, “*in the community, but…trained (with) some kind of public health background… they might be working with churches… native speakers.”* Their overall role was explained as helping patients navigate the complex US healthcare system, which many described as broken.

Specific roles of navigators included helping patients find, access, afford and get medical tests: *“A lot of folks just don't know how …(and) where to go, that's why…xx patient navigator (in) communities are so important…they have social and financial networks to help refer patients…folks don't know where to go, or they have 2 jobs, and they can't take time off work.”*

Navigators also helped patients navigate fears around “scary results:” “*I have stories from lay navigators in Alabama who are like, you know, it takes someone a year to get talked into going to get cancer screening because they're scared of what they're going to find, and I have to go with them. Hold their hand.”*

Bright spots described navigators helping patients access and start treatment quickly, which was perceived as driving improved health outcomes: *“There was a great study published at ASCO… where they had ties between timeliness to help a patient… from symptom onset to diagnosis, from diagnosis to first treatment, from first treatment to…navigation touch points and outcomes were improved every time you could decrease the time to the change.”*

Once diagnosed, navigators also help patients find and enroll in open trials. Patients were described as “unaware” of and “not offered” trial participation opportunities, gaps navigators successfully can bridge:

*“So… talking to various groups of racial and ethnic minorities, the common feedback is not that they didn't want to participate, or were afraid to participate, or didn't understand the study, but mostly that it was not offered to them. That's the part that we're really trying to focus on... You… need that component of extra support…. At a site and at a patient level… like nurse navigators, we're learning, they play a very important role….”*

Bright spots described preferring to partner with navigators to increase awareness about trial opportunities rather than directly distributing information themselves: *“what we are trying to do is partner with sites to distribute that, so that we are not necessarily reaching out purely as a sponsor, but… through navigation…. we're kind of trying to share with partners rather than directly from (our company).”*

Further, bright spots invest heavily in strengthening the infrastructure for navigators and community health workers, recognizing gaps in their abilities to support clinical trials. Some bright spots report conducting formal landscape assessments of navigators, community health workers, and peer support infrastructure across the US. healthcare system, finding very little navigational infrastructure specific to clinical trials. These same bright spots are helping fund the development of more robust navigational services around trials, for their and the industry’s use:

*“…nurse navigators… play a very important role, and we've supported a third-party grant program through the American Cancer Society, which…have been very, very successful…. We support… 14 of the grants and…provide not only for the head count for the nurse navigator…but also the… community of support and education… ensuring that those individuals… placed in those roles have the right resources and tools to support patients and provide that extra level of support throughout this study course. Not just… once they're enrolled, but also for adherence purposes… requirements of the protocol…. our desire (is) to try to provide that resource across oncology research centers.”*

One bright spot described creating a patient navigation program for decentralized trials which provides logistical, transportation and linguistic support to enhance patient participation. Another embed community health workers within local health systems to serve as bridges in trial recruitment: *“We funded the hiring of community health workers in partnership with federally qualified health centers and trial sites… who are trained to provide education about trials and refer patients who are potentially eligible,”* to trials.

### Clinician level efforts: training to improve referrals

Bright spots develop and implement clinician facing educational programs to improve the referral process, which many described as “tough” because clinicians often do not want to “lose” a patient to the care of investigators. As such, programs first and foremost educate on “*why inclusion is important, and referral (to clinical trials) earlier is important.”* To this end one respondent shared, “*They (clinicians) want to take care of their patients until they feel they don't have any other options, and they're referring patients too late to clinical trials, they're not qualifying, or they're too sick or comorbidities are high.”* Early referral was described as important for trial eligibility, given trials often exclude patients who are very sick and or have many co-morbidities. (Fig. 2, Table 3).

**Table 3:** Clinician level facilitators for representative research used by bright spots

Facilitators	Study-Derived Interpretative Conceptualization	Exemplar Quotes
Training	Educational programs provide opportunities for ethical persuasion, that is arguments for why inclusion and referral to clinical trials earlier rather than later are important– and how to talk about and introduce clinical trials to their patients. They also target potential biases clinicians may have in offering enrollment opportunities to patients	• “We interviewed this woman with lung cancer and thankfully, a year after surgery she's lung cancer free. But she's like, why didn't I learn about a clinical trial earlier? I heard about it 4 months after my lumpectomy. Why is it not in general practitioners' office? How come? We don't know that this is available. I think teaching physicians how to talk about it” is important.” • “They (clinicians) want to take care of their patients until they feel they don't have any other options, and they're referring patients too late to clinical trials, they're not qualifying, or they're too sick or comorbidities are high.” • “…you had to think about how you were going to ask that question.… People would get offended if it sounded like they were treating patients differently. When you can tell largely, patients are being treated differently, and unfortunately have lots of horror stories from especially our Black women in diagnosis. So, how to build cultural humility, improve trust in communities, how to help people understand where clinical trials potentially fit in their pathway.” • “We have an amazing …co-created healthcare provider area education program that's external, only an hour and a half…. It's a training… how do we get people (to) do training?”

Programs also offer guidance for clinicians on how to talk about and introduce clinical trials with their patients. One respondent shared they identified this as a critical need after they interviewed various patients about participation barriers:

*“We interviewed this woman with lung cancer and thankfully, a year after surgery she's lung cancer free. But she's like, why didn't I learn about a clinical trial earlier? I heard about it 4 months after my lumpectomy. Why is it not in general practitioners' office?...I think teaching physicians how to talk about it” is important.*

Clinician programs further target potential clinician biases in offering enrollment opportunities. Here, bright spots described trying to offer such programs carefully, in ways that do not to “offend”:

*“…you had to think about how you were going to ask that question.… People would get offended if it sounded like they were treating patients differently. When you can tell largely, patients are being treated differently, and unfortunately have lots of horror stories from especially our black women in diagnosis. So, how to build cultural humility, improve trust in communities, how to help people understand where clinical trials potentially fit in their pathway.*

Despite substantial investments in educational programs*,* bright spots expressed difficulty in getting clinicians to complete training programs: *“We have an amazing …co-created healthcare provider area education program that's external, only an hour and a half…. It's a training… how do we get people (to) do training?”.*

### Trial and site level facilitators

Facilitators operating at the trial site and protocol levels were multifaceted, encompassing four main strategies: (1) enhanced leveraging of existing site networks along with capacity building to expand into new site locations, (2) co-development of protocols with patients, (3) simplification and decentralization of trials to improve patient access, and early and sustained engagement with communities surrounding trial sites. (Fig. 2, Table 4).

**Table 4:** Trial and site level facilitators for representative research used by bright spots

Facilitators	Study-Derived Interpretative Conceptualization	Exemplar Quotes
1. Expansion and leverage of sites and site networks	Bright spots identify and expand into new trial sites. They leverage infrastructure and networks of sites they already use to expand into new areas	• “… we're trying to not always go back to the sites we've used always in the past.” • “We’re try(ing) to stand up if you will new sets of sites that have more of a diverse population are caring for more diverse population, and where, you know previously… studies…frankly, weren't conducted… many of these sit right with an affiliation to the NCI designated cancer center. So, for example, you know you have, (AMC), where we've done many studies over many years. But now we're bringing in satellite Hospital x, which is, of course, affiliated with the AMC but prior to…our efforts (has) really never had access … to the same clinical trials as Emory. Literally, they're a mile and a half from each other, and yet, you know, they've been… a wall between the 2 centers. So we're working toward bringing in those community affiliated networks where, where possible…. And like, I said, every NCI designate cancer center has a satellite hospital and so we're trying to now tap into those.”
2. Co-development of trial protocols with patients	Bright spots create and maintain standing patient committees to review and co-develop trial protocols, including consent processes, eligibility criteria, participation barriers and mitigation strategies, unnecessary data collection and essential data points. Patient committees also help recruit and enroll patients and connect them with trial navigators Committees are comprised of general patients, patients organized by disease groups, and representing specific demographic groups. Each group has their own separate committee	• Bright spots described patient committees as, “really help(ing) us reduce protocol complexity, and it's been very successful.” • “We do have patient panels that review our protocols… we go to a Patient Engagement Council as well to understand their concerns. So, we have all of that before we finalize the protocol…”
3. Simplifying and decentralizing trials	3.1 Streamlining data collection In protocol development, bright spots systematically employ efforts to minimize unnecessary data collection 3.2 Flexible and decentralized designs Bright spots invest in decentralized trial efforts and use virtual contract research organizations to reduce site visit burdens and requirements Broadening eligibility criteria Bright spots have efforts to broaden trial eligibility	• “we try to look at the protocol…to minimize burden… minimized data collection…to only the essential… endpoints that are important for approval, key efficacy and safety endpoints. Also trying to be as broad as possible in the eligibility population. So, take away the things that used to carry over. You know, prior history of certain diseases… if it's under control…there's no biological reason for why that could… affect the study outcome, we then take those out. So, we have a much more… open, inclusive approach…We're reviewing protocols with an eye toward, you know, less data collection and broader eligibility criteria.”
4. Beyond transaction: Early and Sustained Ethical Engagement with Site Communities	Bright spots are intentional about how they engage with patients and the communities around sites. They avoid parachuting into a community just to recruit, and first assess how they can support the community in ways beyond opening a trial site	• “I…think there's been a big focus (on) how we engage with patients and how we actually bring forward the opportunity of a clinical trial like, how do we?” • “…we have to be…a good partner in the community, so that we are not only showing up with questions or pushing information when there's a clinical trial that we are looking to recruit for, because that is a transactional relationship. So really trying to balance this. Obviously, I sit…within clinical development and operations. And so our main mission is to conduct research and run our clinical trials. But…we need to make sure we pay attention to… foundational relationship building. And how do we show up in a way that is genuine and not showing up just to talk, but to listen • “We’re not going to be successful unless we partner with community organizations who are already trusted and have those relationships and credibility in the community. So we have to go through them, and with them, to build that trust and to connect with patients.” • “We’re trying to work with trusted community-based organizations because they already have that connection, right? So it’s not about us coming in and saying, ‘Hey, trust us.’ It’s about us partnering with people who already have that relationship.”
5. Keep US sites open until enrollment targets are met	Bright spots plan and budget for keeping US sites open until enrollment targets for the US are met, even if global goals are hit	• “…When the global target is hit, we don't hold up innovation we'll do the analyses from the protocol that are specified in the global, but we will continue to keep US sites open, just the US sites open until the US enrollment target is met. So just an example of how we plan for a certain US enrollment target from the beginning, and that is a key success metric… we are committed to keep the sites in the US open until that target is met and we've been getting… the financial resources to do that. This means you're over-enrolling, it costs more….”

### Site expansion and network leverage

Bright spots carefully select trial sites based on, “where people live, work, and play” to enhance accessibility. All bright spots and respondents described clear commitments and strategies to identify and expand into new trial sites. As one bright spot shared: *“… we're trying to not always go back to the sites we've used always in the past.”*

Bright spots prioritize leveraging the infrastructure and networks of sites they already use. Here a key strategy includes using all sites affiliated with a National Cancer Institute (NCI) designated Cancer Center, not just the main flagship academic medical center (AMC) site as traditionally prioritized in the past. Bright spots no longer conduct studies only at a flagship AMC hospital, but now also recruit from all satellite and affiliated sites of an AMC, which were described as serving a more diverse patient population who previously could not access sponsors’ trials despite geographic proximity to the flagship AMC’s active site:

*“We’re try(ing) to stand up…new sets of sites that have more of a diverse population… and where, you know previously… studies…frankly, weren't conducted… many of these sit right with an affiliation to the NCI designated cancer center. So, for example, you know you have, (a large AMC), where we've done many studies over many years. But now we're bringing in (the AMC) satellite hospital, which is, of course, affiliated with (the large AMC) but prior to…our efforts (has) really never had access … to the same clinical trials…Literally, they're a mile and a half from each other, and yet…, they've been… a wall between the 2 centers… every NCI designate cancer center has a (satellite) and so we're trying to now tap into those.”*

*Bringing in and leveraging community hospitals and networks affiliated with NCI designated cancer* centers is perceived as helping, “*to bring the trial to the patient instead of…bringing the patient to the trial which creates burden.”* It was also seen as an efficient way to leverage existing infrastructure. As one respondent stated, it, “*leverage(es), you know, with support, extra support, the research infrastructure that's available, but sometimes is not really applied to those community affiliated sites.”*

Bright spots also optimize selection of community care centers for sites, by seeking input from patient groups on where patients were getting care. Bright spots then invite and include those sites in trials. Further, bright spots prioritize enrolling from healthcare centers caring for traditionally underrepresented demographic groups within their target patient populations.

Lastly, bright spots have formal efforts to identify and leverage model sites. Sites enrolling high numbers of minority identifying participants, for example, are labeled as “model sites” and studied to characterize how they achieve success for replication across all their company sites. This was done at Veterans Affairs (VA) sites, for instance. If a VA site consistently recruited and retained patients identifying as a particular demographic better than a peer VA site serving a similar population, the sponsor studied how they did it and then supported their underperforming VA sites to replicate the successful strategies of the high performing site(s). The model site often actively helped develop the capacity of the underperforming or new surrounding sites.

### Standing patient committees and co-development of trial protocols

Bright spots maintain numerous standing patient committees to review and co-develop trial protocols. Committees are comprised of general patients, patients organized by disease groups, and patients representing specific demographic groups, such as women, LatinX or older adult individuals, respectively. Each group has their own separate committee.

Committees and patients review all aspects of trial protocols, including consent processes, eligibility criteria, participation barriers and mitigation strategies, unnecessary data collection and essential datapoints. Bright spots described receiving valuable input on all protocol aspects from patients, which is gathered before a protocol is finalized, not after a trial has trouble meeting recruitment goals: *“we…have patient panels that review our protocols… …we…take patient feedback before…we finalize the protocol.”*

Additionally, committees interact with patients in the recruitment and enrollment process. Committee members help recruit and interview patients. They assist in walking patients through trial protocols and through the consent processes. They speak with patients about participation barriers and help them find support for mitigation. Committee members also facilitate identification of trial coordinators to help with site enrollment. Bright spots described these committees as, “*really help(ing) us reduce protocol complexity, and it's been very successful.”*

### Simplifying and decentralizing trial design to improve patient access

Bright spot trial teams consistently described efforts to simplify trial protocols and enhance accessibility for patients by minimizing unnecessary data collection, adopting decentralized designs, and broadening eligibility criteria.

### Streamlining data collection

Bright spot teams systematically minimized unnecessary data collection during protocol development. One team described a deliberate shift toward focusing *“on endpoints… important for approval, eliminating non-essential safety and efficacy endpoints. Previously endpoints were often as broad as possible.”* This disciplined approach to protocol design was widely reported as a change from prior practices that overburdened sites and patients without adding scientific value.

### Flexible and decentralized designs

Bright spots also invested in decentralized and flexible trial designs, often leveraging virtual contract research organizations (CROs). Teams used digital, remote, and hybrid data collection models to reduce the number of required site visits and mitigate logistical participation barriers, especially those related to geography and transportation. Remote monitoring technologies, such as continuous glucose monitors, were cited as examples of balancing safety oversight with patient convenience.

### Broadening eligibility criteria

Efforts to eliminate unnecessary protocol exclusions and expand eligibility criteria were another consistent theme among bright spot trial teams, particularly those that disproportionately affect underrepresented patients. Common examples included removing blanket exclusions for specific comorbidities:

*“we try to look at the protocol… to be as broad as possible in the eligibility population. So, take away the things that used to carry over. You know, prior history of certain diseases… if it's under control…there's no biological reason for why that could… affect the study outcome, we then take those out.*

Additional considerations included relaxing thresholds for body mass index, blood pressure, or certain genetic mutations when clinically appropriate. Bright spot teams recognized that these changes, alongside reduced visit burdens and simplified data collection, improved both eligibility and retention for patients with complex conditions. Reducing unnecessary genetic testing related exclusions was perceived as important, to bright spot teams, to reduce barriers for uninsured or underinsured patients and those treated at community hospitals, where testing may not be routinely available. In several cases, sponsors directly addressed this structural access barrier by covering the cost of required genetic or biomarker testing.

### Beyond transaction: early and sustained ethical engagement with trial site communities

Bright spots emphasized ethical, long-term engagement with the communities surrounding trial sites. As one team member noted, “There’s been a big focus (on) how we engage with patients and how we actually bring forward the opportunity of a clinical trial.” Rather than “parachuting in” solely to recruit participants, bright spot trial teams prioritized sustained, reciprocal relationships with community partners that avoid overly utilitarian transactional relationships:

*“We have to be… a good partner in the community, so that we are not only showing up with questions or pushing information when there’s a clinical trial that we are looking to recruit for, because that is a transactional relationship.… We need to make sure we pay attention to… foundational relationship building…. showing up not just to talk, but to listen.”*

Before initiating recruitment, bright spots commonly conducted “trust-building sessions” and assessed how to support the community beyond trial operations. Many collaborated with faith-based organizations, such as Black church leaders, and local community groups to strengthen credibility, mutually beneficial relationships, and communication, before launching trials. These organizations, already trusted within their communities, became key partners in patient outreach and education.

### Internally focused company-level efforts

Across bright spots, we identified five strategies or factors for advancing representative clinical trial enrollment (Fig. 2, Table 5):

1. Organizational and leadership commitment,
2. Clear corporate accountability goals with real-time monitoring,
3. Employee education and training,
4. Incentives and rewards for performance, and
5. Minimizing reliance on vendors

**Table 5:** Internally focused company level facilitators for representative research used by bright spots

Facilitators	Study-Derived Interpretative Conceptualization	Exemplar Quotes
1. Known commitment by leadership	Chief Diversity Officer oversees representation efforts and reports performance to the CEO	*“A key to our success in getting traction at all levels of leadership is that our CEO…formed the …Chief Diversity Office that reported directly to him… all 150 officers in this company have actions in all (three) pillars (belonging, inclusive research, and health equity) and they are public…visible to our employees.”*
2. Dashboarding: Clear goals and real-time performance monitoring	Bright spots established explicit representation goals and tracked progress through enterprise dashboards	“Any big companies have these goals around first patient in for a molecule. First data report out to move to late stage, first scrap data for launch, last patient in for the last program for pivotal, filing a drug right there. There might be many things that make up being a corporate goal study. So, if you add, I want this ABC study to have a corporate goal (around representation) they're going to meet their representation goals.”
3. Employee education	Bright spots have internal employee education and training to improve company culture, commitment, and implementation of representation goals. Programs explain why representation is important and how to achieve it	
4. Incentives and rewards	Bright spots reported formal recognition programs tied to health equity and inclusive research performance, with multi-tiered awards and public acknowledgment by senior leadership In several organizations, financial incentives were tied to representation goals	• “… This year we developed inclusive research, health, equity, excellence program, where we recognized all employees that are making a move in around health equity…. we have over 300 recognitions already. It's real time, you get a LinkedIn certificate at a certain level.…. there's 3 levels, and the third level gets officer attention. There’s stuff going on where this counts from a recognition level.” • “It’s huge because it’s tied to everybody’s bonus.” • “Let's just say there's 20 corporate goal studies, maybe there's only 5 that have their goal and maybe 15 of the 20 studies need their goal for us to get the multiplier. …. Whatever committee that makes a decision of whether your protocol goes, or your program molecule goes forward, that visibility on your diversity plans…is needed…you are bringing that data to your leaders quarterly. That's what we're doing.” • “Talk of corporate goals, you could call that an incentive or a carrot or a stick, right? If you meet the goal you, you've changed the world, you're going to be a leader in this work. If you don’t, everybody in the company's going to know. So, it's like you failed right? You failed on a scale across company….”
5. Minimizing reliance on vendors	Bright spots consistently avoided outsourcing representation strategies to vendors, citing poor past experiences with “one-size-fits-all” approaches	• “So, you really must understand at least, you know, if you're involving the NCI designated cancer centers and their community affiliated networks, you have to really get down to the detail of how they're organized. Is it one… central budget, IRB, one EMR and then, you can plan accordingly. Or is it still very loosely connected and you can't leverage a lot of those centralized resources…. we were hoping… that we could apply a broad set of initiatives, and that would work everywhere but that's just not the case.” • “That cookie-cutter vendor approach costs a lot of money but doesn’t really move the needle.”

### Organizational and leadership commitment

When asked about the importance of advancing representation in clinical trial enrollment, all participants responded it is a known priority for their company. Justifications for the priority included seeing it as elemental for fostering patient and clinician trust and confidence in medical products and research finding, for patients to have fair opportunities to participate in research and access its benefits, for good science and good business, for enabling the collection of better and more generalizable product safety and efficacy data, and for health equity. Very plainly, respondents perceived it as the right thing to do and a core company commitment.

While all agreed on the importance of representation in research, one respondent described the goal as important, but not as important as speed and safety in early phase clinical trial objectives. This same respondent expressed that representation becomes internally valued and prioritized when a company identifies a compound as likely to be commercially successful and submitted for FDA approval:

*“(It) becomes more important as you get closer…to hav(ing) a drug that's going to be approvable right? Then you start to say, do you have that right balance? That's why you do randomized trials later… the more you know about the expectations for diversity requirements before you start that pivotal trial the better. And the less I think you are going to be in a situation where you have to run another trial after approval in order to gather the data that the FDA wants, and that has varying challenges sometimes.”*

Interestingly, while all bright spots described their companies having strong commitments to representative research, many described company-wide efforts as emergent and volunteer-driven. These grassroots initiatives often began with informal working groups led by motivated employees, especially within oncology divisions: *“it's actually just this voluntary group of people who are interested in (this) space, and those numbers are quite high, and there's a big interest…we’ve got the sort of ground swell.”* Most bright spots described “formally” beginning and growing their initiatives relatively recently, during the pandemic, in late 2020 and early 2021, supported by enhanced telehealth infrastructure during the pandemic.

One bright spot reported a fully institutionalized governance structure for inclusive research, formalized in 2017, with efforts led by an enterprise-wide Chief Diversity Office reporting directly to the CEO. Leadership accountability extended across all therapeutic areas, with public reporting of officer-level representation goals:

*“A key to our success in getting traction at all levels of leadership is that our CEO…formed the …Chief Diversity Office that reported directly to him… all 150 officers in this company have actions in all (three) pillars (belonging, inclusive research, and health equity) and they are public…visible to our employees.”*

This organization, which also implemented all five company-level strategies, sponsored the largest number of bright spot trials in our sample.

### Dashboarding: clear goals and real time performance monitoring

Bright spots’ commitments to conducting representative cancer research are backed by clear corporate goals and real time progress tracking enabled by enterprise dashboards. Dashboards were described as elevating representation to the level of other key corporate metrics (e.g., “first patient in,” “regulatory submission,” “data lock”). As one participant explained, adding representation goals to a dashboard makes them a corporate goal, so they get met:

*“Any big companies have these goals around first patient in for a molecule. First data report out to move to late stage, first scrap data for launch, last patient in for the last program for pivotal, filing a drug right there. There might be many things that make up being a corporate goal study. So, if you add, I want this study to have a corporate goal (around representation) they're going to meet their representation goals.”*

Dashboard data enabled real-time transparent performance monitoring and timely course correction companywide, as well as by study, region, and therapeutic area:

*“And they're saying, where have we improved? Where have you not? Where do we move in the needle or where do we not? They'll look quarterly across studies, they look at US only, they'll look at global, we can look by study. So those governance committees requiring quarterly reports, that is actionable, and people can understand where they are. We created a…dashboard, so anybody at any time can go in and see what any study is doing. Not at the end when you got the CSR tables, but you know at first patient in, monthly, in quarterly.”*

### Incentives and rewards

Dashboards also facilitated recognition and reward of high performers. Bright spots reported formal recognition programs tied to health equity and inclusive research performance, with multi-tiered awards and public acknowledgment by senior leadership. One company’s inclusive research excellence program recognized over 300 employees through real-time digital certificates and officer-level commendations:

*“… This year we developed inclusive research, health, equity, excellence program, where we recognized all employees that are making a move in around health equity…. we have over 300 recognitions already. It's real time, you get a LinkedIn certificate at a certain level.…. there's 3 levels, and the third level gets officer attention. There’s stuff going on where this counts from a recognition level.”*

In several organizations, financial incentives were tied to representation goals: *“It’s huge because it’s tied to everybody’s bonus.”*

Conversely, underperforming trials risked reputational and funding consequences. As one respondent described, diversity metrics were reviewed at portfolio governance meetings, and trials failing to meet goals could affect advancement of the molecule to later phases:

*“Let's just say there's 20 corporate goal studies, maybe there's only 5 that have their goal and maybe 15 of the 20 studies need their goal for us to get the multiplier. …. Whatever committee that makes a decision of whether your protocol goes, or your program molecule goes forward, that visibility on your diversity plans…is needed…you are bringing that data to your leaders quarterly. That's what we're doing.”*

Transparency around performance created what one participant described as a “carrot or stick” culture: *“If you meet the goal, you’re a leader; if you don’t, everybody knows.”* Another shared:

*“Talk of corporate goals, you could call that an incentive or a carrot or a stick, right? If you meet the goal you, you've changed the world, you're going to be a leader in this work. If you don’t, everybody in the company's going to know. So, it's like you failed right? You failed on a scale across company….”*

### Employee education and training

Bright spots invested in structured education programs to strengthen company culture and operational capacity around representation goals. Training initiatives for employees emphasized both *why* representation matters (e.g., for scientific rigor, business success, and patient equity) and *how* to achieve it. Programs taught teams to identify sites with patient populations aligned to disease demographics and to apply epidemiologic and incidence data to guide recruitment planning. These efforts were viewed as essential for embedding inclusive research practices across the organization.

### Minimizing reliance on vendors

Bright spots consistently described avoiding outsourcing representation strategies to vendors, citing poor past experiences with “one-size-fits-all” approaches. They emphasized the need for tailored, site-specific engagement that external partners often failed to provide. As one respondent noted, what works “for Yale wouldn’t work for UCSF…it’s just a different system.” Understanding the structure of site networks, institutional review processes, and community relationships was seen as critical for success, factors vendors often overlooked:

*“So, you really must understand at least, you know, if you're involving the NCI designated cancer centers and their community affiliated networks, you have to really get down to the detail of how they're organized. Is it one… central budget, IRB, one EMR (electronic medical record) and then, you can plan accordingly. Or is it still very loosely connected and you can't leverage a lot of those centralized resources…. we were hoping… that we could apply a broad set of initiatives, and that would work everywhere but that's just not the case.”*

Multiple bright spots described vendors as opportunist, taking advantage of a need without proper tools, expensive, and ineffective: *“That cookie-cutter vendor approach costs a lot of money but doesn’t really move the needle.”* Instead, many bright spots invested in in-house navigators and site relationship managers who could build sustained, localized engagement and address patient-level barriers directly.

### Federal and cross-industry collaboration efforts

#### Requests for enhanced clarity on adequate representation in FDA guidance: What are the goal posts and for which trials?

Though generally positive about FDA guidance, bright spots also requested additional guidance, noting, “*there’s still ambiguity in the FDA guidance.”* While FDA guidance documents generally talk about applying representation recommendations across a development plan including early phase trials if appropriate, a bright spot asked: *"What studies does this (guidance) apply to? Does it apply to registrational studies only? Is it for pivotal trials? What about early phase? What about post-marketing studies? What do you mean by ‘diverse’?".*

Another critiqued variability in feedback across the FDA as an organization when discussing enrollment objectives for trials with them and asked for more uniform and clear objectives particularly around which groups should be prioritized in representation efforts. They also requested greater consistency in how guidance is interpreted and applied by the FDA to individual trials.

When asked how to define adequate representation, one bright spot responded, *“I mean, I think that's a great question. That's a million-dollar question.”* Another responded that it could vary by trial phase: *“I think it depends on the stage of the trial….as you get further in development, I think it becomes much more clear what patient populations you're targeting and then we're now setting those targets as part of our diversity plan….”*

Despite respondents’ uncertainty about definitions of adequate trial representation, and FDA priorities in this regard, all bright spots described conceptualizing adequate representation as when enrollment demographics mirror disease incidence or prevalence across different groups:

*“Census data, treatment data, again, we would leverage the data sources that we have available to sort of say… what is the representative population that gets treated for that? Then how would we make sure that our trial is focusing on any special populations that may be represented there, and setting targets for that early.”*

Bright spots also expressed challenges in using global trial enrollment to mirror US epidemiological data. Most trials enroll globally, and it was unclear to many whether global or US enrollment should mirror the US patient demographics to achieve representation. Often companies lack real world incidence and prevalence data broken down by certain demographic groups for many indications, making it challenging to determine adequate representation. In this case, some revert to using proxy data and census data to set enrollment goals. While some bright spots consider a trial successfully enrolled only when it represents both global and U.S. enrollment targets, others focus more narrowly on US representation. Some bright spots keep U.S. sites open beyond global enrollment completion dates to meet representation goals​.

“…When the global target is hit, we don't hold up innovation we'll do the analyses from the protocol that are specified in the global, but we will continue to keep US sites open, just the US sites open until the US enrollment target is met. So just an example of how we plan for a certain US enrollment target from the beginning, and that is a key success metric… we are committed to keep the sites in the US open until that target is met and we've been getting… the financial resources to do that. This means you're over-enrolling, it costs more….”

#### Efforts to end trial participation expense reimbursements as taxable income and support participation of under and uninsured patients

Bright spots are working to change US federal policy in several ways. Many described working to end the classification of trial participant expense reimbursements as taxable income, at certain thresholds. Bright spots portrayed taxation as a disincentive for trial participation and acceptance of needed financial support.

Further, bright spots described collaborations to support participation of under and uninsured patients in trials, a complicated task given the general lack of or less access to care for these populations.

#### Support of FDA guidance

All bright spot teams expressed support for and welcomed FDA efforts to enroll a representative population in clinical research, describing them as helping advance consistency across the industry and across FDA reviews and efforts. Bright spot teams also agreed that FDA action was necessary to incentivize and drive internal company change and commitment, perceiving other companies as unlikely to voluntarily prioritize and make progress without FDA efforts to keep them accountable:

*“I think that we've seen that without being more prescriptive, we're not enhancing diversity…. my personal view is, making sure that we are being held accountable for what we're doing, both from guidance and then law, is actually essential to move the needle forward…. It makes people listen and take action in a different way. So, before it was important and now… it's elevated the conversation to where it should be that it's essential that we look at it, and very methodically and intentionally try to have our trials be representative.”*

#### Implementation checklist

The strategies described by bright spots as successful for enhancing participation of patient groups in cancer-related pivotal trials can be categorized into a 16-point implementation checklist: 6 strategies for implementation before a trial begins and 10 occurring during trial design and implementation (Table 6).

**Table 6:** Implementation checklist

Strategy	Drug Development Stage/Trial Phase	Responsible Functional Owner(s)	Key Resource Requirements	Key Performance Indicator (KPI)
Before the trial, capacity building				
1. Co-develop community-informed culturally tailored educational programs with patients and community partners about clinical trials	Pre-trial engagement; recruitment; ongoing portfolio-level	Medical Affairs; Patient Engagement; Health Equity teams	Community advisory boards; multi-lingual materials; translation services; community partnerships; grant funding	Patient awareness metrics; referral rates from community channels; engagement reach
2. Conduct early, sustained, non transactional engagement with communities around sites (≥ 6 months prior to activation)	Pre-trial engagement; recruitment	Community Engagement; Site Management; Medical Affairs	Partnerships with faith-based organizations; community listening sessions; outreach funding	Number and duration of community partnerships; community referral rates; trust/engagement indicators
3. Deploy patient-facing trial websites with prescreening and direct connection to enrolling sites	Trial planning; Recruitment phase; Portfolio infrastructure	Digital Health; Clinical Operations; Patient Engagement	Website design; digital prescreening tools; vendor support for digital platforms; patient UX testing	Website conversion rate (visit → site contact); prescreen completion rate
4. Fund and deploy patient navigators and community health workers, including bilingual and community-embedded roles	Diagnosis-to-enrollment pathway; recruitment and retention	Site Management; Medical Affairs; Community Engagement teams	Navigator staffing; training; grants to sites/community organizations; navigation infrastructure	Screening-to-enrollment rate; retention/adherence rates
5. Train clinicians on early referral, culturally competent communication, and equitable trial offering practices	Pre-trial engagement; recruitment	Pre-trial engagement; recruitment; Medical Affairs; Clinical Development; Professional Education	Continuing medical education modules; clinician toolkits; referral pathway education	Referral timing (diagnosis → referral); proportion of eligible patients offered trials
6. Establish executive-level accountability and governance structures for representative research	Enterprise governance across development pipeline	Executive leadership; Chief Diversity or Health Equity offices	Governance frameworks; leadership KPIs; reporting structures; performance dashboarding; employee incentive structures	Inclusion of representation in corporate goals; leadership performance evaluations tied to outcomes
Trial planning and design phase				
1. Prospectively budget and pre-fund participant support (transportation, lodging, meals, dependent care), minimizing reimbursement burden	Trial planning; protocol budgeting; Phase II–III pivotal trials	Clinical Operations; Finance; Patient Engagement	Dedicated budget line items; prepaid debit card systems; reimbursement infrastructure; patient support staff	% participants receiving support; dropout rate due to financial barriers
2. Expand trial sites to include community hospitals and satellite networks affiliated with NCI-designated cancer centers	Trial planning; site selection; recruitment; Phase II–III pivotal trials	Clinical Operations; Site Feasibility; Site Management	Expanded feasibility analytics; onboarding support for community sites; Network contracting support; centralized IRB and EMR integration planning	% sites that are community-based; % sites participating from network affiliated with NCI designated cancer center; % sites that are community-based; enrollment contribution by site type
3. Engage standing patient advisory committees to co-develop protocols	Protocol development; trial design	Clinical Development; Medical Affairs; Patient Engagement	Patient advisory boards; meeting facilitation; compensation for patient advisors	Number of protocol changes informed by patient input; recruitment performance vs projections
4. Simplify protocols by prioritizing essential endpoints and minimizing non-essential procedures	Protocol design; regulatory planning	Clinical Development; Biostatistics; Regulatory Affairs	Protocol governance processes; endpoint prioritization frameworks	Number of protocol procedures/visits; site burden metrics; enrollment speed
5. Implement decentralized and hybrid trial designs to reduce visit burden and geographic barriers	Trial design and implementation	Clinical Operations; Digital Health teams; CRO oversight	Remote monitoring technologies; telehealth platforms; decentralized trial vendors	Average number of required site visits; geographic diversity of participants
6. Broaden eligibility criteria by removing non–scientifically justified exclusions (e.g., stable comorbidities)	Protocol design; regulatory review	Clinical Development; Regulatory Affairs; Medical Affairs	Eligibility review frameworks; epidemiologic data; clinical justification processes; biomarker testing support	% screened patients eligible; demographic diversity of eligible pool
7. Provide sponsor-funded biomarker/genetic testing when needed for eligibility	Screening phase; recruitment	Clinical Operations; Medical Affairs	Diagnostic testing funding; laboratory partnerships	% patients receiving testing support; screening completion rate
8. Set explicit representation targets aligned with epidemiologic data and monitor performance via real-time dashboards	Portfolio oversight across all trial phases	Clinical Operations leadership; Data Analytics; Governance committees	Real-time enrollment dashboards; analytics infrastructure	Enrollment vs epidemiologic benchmarks (by race/ethnicity); time to target attainment
9. Develop internal, site-specific relationship management capabilities rather than relying on generic vendors	Site selection; recruitment; site engagement	Clinical Operations; Site Management	Dedicated relationship managers; site engagement budgets	Site performance variability; enrollment rates by site
10. Identify high-performing “model sites” and systematically replicate their practices across the network	Ongoing trial monitoring; portfolio learning	Clinical Operations; Data Analytics teams	Site performance analytics; peer-learning programs; replication toolkits	Performance improvement across sites over time; reduction in underperforming sites

## Discussion

In this qualitative study of “bright spot” teams leading oncology trials that supported FDA new drug approvals between 2012 and 2021 that achieved adequate enrollment of Black or LatinX-identifying patients relative to disease burden, we identified a coherent set of strategies and factors associated with representative enrollment. Across large pharmaceutical sponsors, facilitators clustered at the patient/community, clinician, trial/site, company, and policy levels, emphasizing practical steps that reduce burden, expand access, and embed accountability.

At the trial and site levels, three strategies recurred: (1) streamlining protocols to minimize nonessential data collection, (2) adopting decentralized or hybrid designs to reduce visit burden and logistical barriers, and (3) broadening eligibility criteria, including removal of unnecessary exclusions for comorbidities or genetic testing requirements. Sponsors often covered biomarker testing costs for underinsured patients and used navigators to support screening and retention. Teams emphasized early, sustained partnerships with community organizations to build trust and avoid transactional and “parachute” recruitment. At the company level, bright spots described reinforcing mechanisms that institutionalized inclusion: employee education that clarified both the rationale and methods for representative research; enterprise dashboards tracking progress toward explicit representation goals; recognition and financial incentives tied to performance; and deliberate movement away from generic vendor-led approaches toward internally managed, site-specific engagement. Most organizations reported early-stage, volunteer-driven efforts, though one early adopter implemented enterprise-wide governance with CEO accountability, coinciding with a larger number of bright spot trials.

These findings extend prior literature which largely catalogues barriers to representative enrollment, by offering solutions. Findings also add operational specificity to policy guidance, emphasizing protocol parsimony, prospective budgeting for participant support, real-time performance monitoring, and integration of representation metrics into corporate objectives. Our findings support the importance of measurement as a tool for accountability and the old adage, what doesn’t get measured, doesn’t get done. Additionally our findings suggest pairing broader eligibility with operational and patient supports such as trial navigators and expense mitigation supports representative trial enrollment. Participants welcomed regulatory attention to representation planning and clarity on scope (e.g., phase applicability), priority populations, and acceptable data sources for setting enrollment targets. Given that many pivotal trials enroll globally while representation targets are often US-specific, harmonized expectations and practical approaches (e.g., keeping US sites open longer; prespecified regional quotas) may reduce uncertainty. Guidance on managing over-representation versus under-representation in multinational programs may also be needed. Industry efforts to classify participant reimbursements as non-taxable and to address insurance gaps were viewed as important levers to lower financial barriers.

This study has limitations. Findings rely on self-report and may reflect social desirability bias. As a qualitative study of high-performing bright spot trials, findings are intended to generate insights into practices used in successful cases not to provide generalizable estimates of sponsor behavior across all oncology trials. The focus on oncology and large companies may describe experiences most relevant to similar companies. Qualitative research is well suited to richly describe complex phenomena within their context. This study further explains the contextual environment and activities that exist among bright spot sponsors. Identification of critical similarities as well as differences across companies with different cultures and operations, however, supports the credibility of our findings among the sample. In December 2025, the FDA finalized updated guidance emphasizing enhanced participation and representativeness in clinical trials, including expectations around prospective enrollment planning and justification of eligibility criteria. Although the terminology and scope evolved, the core principles align with many strategies identified in our interviews. Because our interviews predated this final guidance, sponsors’ approaches reflect practices under earlier regulatory expectations. Because we did not quantitatively link practices to outcomes, causal inference is not possible. Future research should test these hypotheses across settings and evaluate the effectiveness and cost of the identified strategies.

## Conclusion

Sponsors that achieved representative enrollment in pivotal trials of cancer therapeutics shared a common operational playbook: simplify what is asked of patients and sites, bring trials closer to where people receive care, widen the doors of eligibility, invest in trusted relationships, and hold trial teams accountable with timely data. Translating these practices from bright spots to standard practice, supported by clearer regulatory expectations, stronger community infrastructure, and rigorous evaluation, offers a practical path to more representative and accessible clinical research and evidence.

## Supplementary Information

Supplementary Material 1.

Supplementary Material 2.

## Data Availability

Anonymized and de-identified data are available upon request for research purposes.
