# Supplementary material for: Strategies for enhancing participation of underrepresented patient populations in cancer clinical trials: learning from “bright spots”
Source: BMC Cancer. 2026 Jun 13;26:1009. doi: 10.1186/s12885-026-16181-1 (PMC13501612; doi:10.1186/s12885-026-16181-1)
Supplement: Supplementary file 1 [file 12885_2026_16181_MOESM1_ESM.docx]

OMB Control No: 0910-0847	Expiration Date: 2/28/2026

A Positive Deviance Approach for Representing Women, Older Adults and Patients Identifying as Racial and Ethnic Minorities in Oncology Research

Paperwork Reduction Act Statement: According to the Paperwork Reduction Act of 1995, an agency may not conduct or sponsor, and a person is not required to respond to a collection of information unless it displays a valid OMB control number. The time required to complete this information collection is estimated to average 5 minutes per response, including the time for completing the screen questions, testing the interview link, logging onto the online platform, reviewing instructions, searching existing data sources, gathering, and maintaining the data needed, and completing and reviewing the collection of information.

Send comments regarding this burden estimate or any other aspects of this collection of information, including suggestions for reducing burden to [PRAStaff@fda.hhs.gov](mailto:PRAStaff@fda.hhs.gov)<mailto:>.

The study we are conducting is on behalf of the U.S. Food and Drug Administration (FDA).

**Oral Consent Email Script**

You are being asked to participate in a research study designed to help advance health equity and the inclusion of special populations in cancer drug development.

Our study, “A positive deviance approach to representing women, older adults and patients identifying as racial and ethnic minorities in oncology research,” seeks to improve the inclusion of women, older adults, and minoritized patients in cancer research. Utilizing samples of pivotal trials supporting FDA approval of novel cancer therapeutics from 2012-2021, we analyzed and identified top performing sponsors (“positive deviants”) on enrolling demographically representative trial populations. Trial participant demographics were compared to those of the patients with the disease or condition targeted in a study by constructing a *participant-to-prevalence ratio* (PPR). Studies with at least a PPR of 0.8 were considered to have adequate representation and received a score of 100%. We are now conducting qualitative interviews with staff and administrators that were positive deviants on our measures.

We have identified a study that was conducted by you and your team that reflected strong representative research. Due to your high participant to prevalence score, you are invited to participate in a qualitative interview.

Study participation includes a 1-hour qualitative interview, which will be audio recorded via Zoom, to discuss the major initiatives your group has undertaken to enable representation of women, older adults, and/or Asian, Black and LatinX-identifying patients in trials. The interview will be transcribed by Yale staff. You will also be asked for some basic demographic information. You will not receive payment for your participation.

There are no known risks to you in taking part in this interview.

Our hope is that this research will identify strategies for advancing diversity and adequate representation of women, older adults, and racial and ethnic minoritized patients in oncology clinical research.

Your information collected as a part of this research could be used for future research or distributed to another investigator for future research without additional informed consent from you, only after information that identifies you is removed.

Please understand your participation is voluntary and you have the right to withdraw your consent or discontinue participation at any time without penalty.

This research has been reviewed by the Yale University Institutional Review Board. If you have any concerns, complaints, or general questions about research or your rights as a participant, please contact the Yale Human Investigation Committee at 203-785-4688.

If you have any questions concerning the research study, please contact Jennifer Miller at [Jennifer.e.miller@yale.edu](mailto:Jennifer.e.miller@yale.edu).
