# Supplementary material for: Strategies for enhancing participation of underrepresented patient populations in cancer clinical trials: learning from “bright spots”
Source: BMC Cancer. 2026 Jun 13;26:1009. doi: 10.1186/s12885-026-16181-1 (PMC13501612; doi:10.1186/s12885-026-16181-1)
Supplement: Supplementary file 2 [file 12885_2026_16181_MOESM2_ESM.docx]

OMB Control No.	Expiration Date XX-XX-XXXX

Project Title: A Positive Deviance Approach for Representing Women, Older Adults and Patients Identifying as Racial and Ethnic Minorities in Oncology Research

Paperwork Reduction Act Statement: According to the Paperwork Reduction Act of 1995, an agency may not conduct or sponsor, and a person is not required to respond to a collection of information unless it displays a valid OMB control number. The valid OMB control number for this information collection is 0910-0847. The time required to complete this information collection is estimated to average 50 minutes per response, including the time for completing the screen questions, testing the interview link, logging onto the online platform, reviewing instructions, searching existing data sources, gathering, and maintaining the data needed, and completing and reviewing the collection of information.

Send comments regarding this burden estimate or any other aspects of this collection of information, including suggestions for reducing burden to [PRAStaff@fda.hhs.gov](mailto:PRAStaff@fda.hhs.gov).

The study we are conducting is on behalf of the U.S. Food and Drug Administration (FDA).

**Zoom Interview guide, activities, demographics survey**

**Survey questions (to be administered via an online form at the beginning of the interview)**

1. Familiarity with diversity, equity and inclusion (DEI) challenges and efforts in oncology clinical research (Y/N)

2.  Familiarity with demographic disparities in clinical research across age, sex, and race and ethnicity in clinical research (Y/N)?

3. Improving DEI in clinical trials will improve medicine (Strongly Disagree, Disagree, Neither Disagree nor Agree, Agree, Strongly Agree)

4. Experience with the application of internal policies and procedures to advance diversity, equity and inclusion in clinical research within your company (Y/N)

5. Knowledge of FDA guidance on improving diversity, equity and inclusion in clinical research (novice, advanced beginner, competent, proficient, and expert)

6. List 3 efforts by your company to advance diversity, equity and inclusion in clinical research that you/your team use in practice that you think are promising/ valuable. (Free text)

**Activities and instructions for research team**

1. Ask respondents to complete above survey questions.

2. Ask respondents a “grand tour" question: “In the last 3-5 years, what are the major initiatives your group has undertaken to enable representation of women, older adults and or Asian, Black, LatinX, Indigenous or Native American, and Native Hawaiian and Other Pacific Islanders- identifying patients in trials.”

3. Then, we will move to the particular, and include probes for clarification and additional detail and ask respondents to describe specific instances of difficulty and success in research execution and experiences monitoring progress and sustaining efforts. Interview responses comprising stories will be encouraged.
4. We will conclude the interview by presenting a list of strategies for improving DEI in trials and ask participants to rank the list based on perceived utility. Group brainstorming, will follow, if time allows, to make additions to the list.

**Interview guide**

Starting the discussion

- Thank you for agreeing to participate in this interview.
- Before we start, we have a brief sheet for you to complete to let us know the demographics of discussion participants.
- Thank you for completing the survey.
- Brief introductions by researchers and then invite participants to introduce themselves
- Note that we will be using first names only during the discussions
- Review purpose of study: We are interested in learning about your perspectives and experiences with administering clinical trials, especially among some groups such as older adults and racial and ethnic minoritized people who have not always been included in clinical trials.

Explaining the discussion process

I’d like to cover a few important aspects of the discussion group.

- Participation in the group interview will take 60 minutes.
- While participating in the interview, you will be asked to give your opinion about what has worked and what has not in your team’s/organization’s experiences with clinical trials. We appreciate your candor and honesty.
- There are no right or wrong answers to the questions we pose. We are interested in advancing our understanding of all clinical trials by learning from diverse perspectives.
- This session will be audio- recorded. Is everyone okay with this?
- The consent form was emailed to you. Did you have any questions about the consent form?
- All information obtained about you during the conversation will be held in confidence and accessible only to the research team of this project. We will not collect any information from you that will make it possible to trace your responses back to you. Data will be anonymized and aggregated for reporting purposes.
- Your participation is voluntary. You are free to decide whether you want to participate in this conversation or any individual question. If you do not want to participate, you are free to not answer any question or leave at any time and it will not harm your relationship with Yale University.
- Your perspective is important. So please, say what comes to mind, both positive and negative. Even if you have a dissenting opinion from someone else in the group, we want to hear it.
- We also ask that team members participating in group interviews hold comments shared in this session in confidence.

Does anyone have any questions before we start?

(answer any questions)

Okay, we can jump into our first activity, which is a quick survey with 6 questions (See “**survey questions” above).**

Thank you for completing the survey, let’s jump right into the open discussion questions.

(see “open discussion prompts” below)

**Open discussion prompts:**

1. The FDA has undertaken a series of initiatives to make public and improve the rates of enrollment of populations of patients in clinical trials, including women, older adults and racial and ethnic minoritized populations. What do you think of these initiatives? Is this an important problem to solve?
2. In the last 3-5 years what initiatives has your group undertaken to enable representation of:
   a. Women in trials?
   b. Older adults in trials?
   c. Racial and ethnic minoritized patients in trials? Can you describe from which racial and ethnic groups you have sought greater representation?
   d. Where these initiatives focused on a particular therapeutic area?
3. Can you describe instances of difficulty and success with these initiatives? Why did initiatives work or not work?
4. Can you describe how these initiatives got started (i.e., origin of idea, funding, partners)?
5. More generally, can you describe instances of success in recruiting and retaining:
   a. Women in trials?
   b. Older adults?
   c. Racial and ethnic minoritized patients, in trials?
6. Can you describe instances of difficulty in recruiting and retaining?
   a. Women?
   b. Older adults?
   c. Racial and ethnic minoritized patients in trials?
7. What counts as adequate demographic representation in a clinical trial for your organization/institution?
   a. What are some of the benefits relative to defining adequate demographic representation in this way?
   b. What are some of the drawbacks relative to defining adequate demographic representation in this way?
8. What counts as under-and over- representation in clinical research?
   a. What are some of the benefits to over and under representation?
   b. What are some of the drawbacks relative to over and or under representation in research?
9. Does your company collect demographic data on all trial participants, if so which data and how are data collected? Are demographic data self-reported or self-identified?
10. In what ways is collecting self-identified race or ethnicity data like or different than collecting other demographic data about patients (for example, age).
11. What do you think of current infrastructure, incentives, and governance supporting clinical trial diversity? How do these facilitate or hinder advancing clinical trial diversity?
12. What type of oversight do you think your company or research ecosystem need to raise trial participation of women, older adults and racial and ethnic minoritized patients?
13. What would facilitate your company and the research ecosystem in increasing demographic participation (e.g., incentives, culture)? Does your company work to provide these facilitators, if so how or if no, why not?
14. What systemic barriers does your company or the research ecosystem encounter in increasing demographic participation (e.g., incentives, culture)? Does your company work to overcome these barriers, if so how or if no, why not?
15. What type of support do you think patients (women, older adults and or racial and ethnic minoritized patients) need to increase their trial participation? Does your company work to provide this support, if so how or if no, why not?

**Analysis:**

Content analysis will be performed on verbatim transcripts using a constant comparative approach^1^ to identify distinct themes shared by high-performers on specific representation measures.

**Results:**

1. Identification of generalizable behaviors, strategies and contexts shared by positive deviant sponsors, enabling them to adequately represent women, older adults and patients identifying as racial and ethnic minorities in oncology clinical trials, that can be implemented by the broader research community to produce similar results.
2. Findings will be characterized and disseminated through a series of high impact peer-reviewed journal articles and presentations.
